# Immunogenicity and Complications of the Pentavalent Vaccine in Iranian Children

**DOI:** 10.3389/fped.2021.716779

**Published:** 2021-10-01

**Authors:** Mina Ekrami Noghabi, Mohammad Jafar Saffar, Shaghayegh Rezai, Hana Saffar, Hiva Saffar, Fatemeh Hosseinzadeh, Aliasghar Nadi Ghara, Mohammad Sadegh Rezai

**Affiliations:** ^1^Pediatric Infectious Diseases Research Center, Communicable Diseases Institute, Mazandaran University of Medical Sciences, Sari, Iran; ^2^Department of Microbiology and Virology, Mashhad University of Medical Sciences, Mashhad, Iran; ^3^Anatomical and Clinical Pathology, Department of Pathology, Cancer Institute, Tehran University of Medical Sciences, Tehran, Iran; ^4^Health Science Research Center, Addiction Institute, Mazandaran University of Medical Sciences, Sari, Iran

**Keywords:** immunogenicity, pentavalent, hemophilus influenzae b, pertussis, diphtheria, hepatitis B

## Abstract

**Objective:** Vaccination is one of the most convenient and safe preventive care measures available for children. The Pentavalent vaccine which protects against five major infections including diphtheria, tetanus, pertussis, hepatitis B(HepB) and *Haemophilus influenzae type b*(*Hib*) was added to the Iranian national immunization program in November 2014. This study aimed to determine the Pentavalent vaccine adverse events and immunogenicity in an Iranian children population in Sari, northern Iran.

**Method:** In this descriptive-analytical study, children who were vaccinated with three doses of the Pentavalent vaccine were studied. Two venous blood samples were obtained before the first dose and 4 weeks following the last booster dose. Possible local and systemic complications of the vaccine were recorded until 7 days following vaccination. Antibody titers were measured by quantitative ELISA kits and geometric mean titer(GMT) was calculated for each vaccine component before and after 3 doses of vaccine. Statistical analysis was performed by SPSS 20.0 software and Chi-square and Fisher's exact tests were used for analysis.

**Results:** Immunogenicity of the Pentavalent vaccine for tetanus was 100%(GMT:2.52 Eu/mL, 95%CI: 2.22–2.88), *Hib* 98.7%(GMT:2.44 Eu/mL, 95%CI: 2.06–2.89), HepB 98.7%(GMT:153.54 Eu/mL, 95%CI: 133.73–176.29), diphtheria 93.1%(GMT:0.43 Eu/mL, 95%CI:0.37–0.51) and pertussis were 63.7% (GMT:19.44 Eu/mL, 95%CI:16.42–23.03). The most common systemic complication after vaccination was fever. Also, one infant cried for more than 3 hours after the second dose. Other serious side effects were not observed.

**Conclusion:** The Pentavalent vaccine used in Iran can cause adequate antibody response against diphtheria, tetanus, pertussis, *Hib* and hepatitis B in most cases with minimal side effects. The immunogenicity of this vaccine is significantly lower for pertussis. In this study, no severe complication leading to contraindication to subsequent injections was reported. So, the present policy in replacing triple DTP vaccine with Pentavalent vaccine should be continued in Iran.

## Introduction

Immunization, as a cost-effective and life-saving intervention, is a fundamental component of public health policies for controlling infectious diseases (1, 2). Nowadays, multivalent vaccines like the tetravalent (DTP-*Hib* or DTP–HepB) or the Pentavalent (DTP-HepB-*Hib*) combination vaccines have been added to the immunization program in many countries (3).

The prospect of up to six or seven separate injections at each visit for an infant is stressful for parents (4). Numerous advantages are documented for combined vaccines including decrease in the number of clinic visits, protection against several diseases in one injection, better immunization coverage, simple, easy-to-administer fully liquid formulations, fewer injections and less distress for children, lower shipping and transport costs, fewer syringes and an increase in parental consent (1, 2, 4). Therefore, the addition of antigens to existing vaccines with high coverage is considered an effective and appropriate strategy for protecting society from new diseases (5).

World Health Organization (WHO) has recommended the Expanded Program on Immunization (EPI), which integrates the *Haemophilus influenza type b* (*Hib*) vaccine into the routine pediatric immunization program. The first combination vaccine was used in children in 1940 (6). Five antigens are currently available as a Pentavalent combination vaccine for protection against tuberculosis, diphtheria, tetanus, whooping cough, poliomyelitis, and measles, as well as HepB and *Hib* in the national immunization program in developing countries (7, 8). According to the WHO, vaccines are one of the most powerful tools in public health and more children are now being immunized than ever before (9).

From December 2014, the Pentavalent vaccine was added to the current national vaccination program for Iranian children and is injected according to schedules 2, 4 and 6 months after birth. Public trust in newly introduced vaccines can be strengthened by monitoring vaccine's safety (1). Also, reporting side effects play an important role in assessing the safety of vaccines, especially for newer vaccines that are less commonly used and less experienced. So far, no vaccine has been 100% effective and safe for everyone.

The source of the vaccine used in Iran is the Serum Institute of India. Due to the use of the Pentavalent vaccine in recent years and the lack of accurate information about its immunogenicity, evaluating the antibody titer and immunogenicity of this vaccine and its side effects is necessary. This study aimed to evaluate the immunogenicity of the Pentavalent vaccine and its side effects in Iranian children.

## Materials and Methods

### Study Population

In this cross-sectional descriptive-analytic study, apparently healthy infants of both genders between 42 and 64 days (at the time of first vaccination) referred for the Pentavalent vaccine injection at 2 months of age in Sari city, north of Iran from January to September 2019 were evaluated. Inclusion criteria included delivery at 36–40 weeks of gestational age with a birth weight over 2,500 g and a normal Apgar score of 1 and 5 min with no congenital defects who received all three doses of the Pentavalent vaccine. Infants with moderate to severe disease, known/suspected immune function impairment, previous treatment with a parenteral immunoglobulin preparation and/or blood products, vaccination against *Hib* and/or DTP, history of anaphylaxis or hypersensitivity to any vaccine ingredient, or presence of clinically significant acute infection or acute illness, cerebral-neurological disorders and seizures were excluded.

### Study Design and Vaccination Schedule

Blood samples (3 mL) were collected before the first dose and 4 weeks after the third dose of the Pentavalent vaccine. Infants received the Pentavalent vaccine at 2, 4, and 6 months of age. One vaccinator performed the vaccinations throughout the study, and all three doses were administered by the same trained and qualified vaccinator.

Each 0.5 mL dose of the Pentavalent vaccine (DTaP_5_-IPV-*Hib*) manufactured by the Serum Institute of India contained a combination of ≥30 IU diphtheria toxoid, ≥60 IU tetanus toxoid, ≥4 IU inactivated cellular *Bordetella pertussis*, 10 mg *Hib* polyribosylribitol phosphate (PRP) capsular polysaccharide conjugated to 10 μg tetanus toxoid adsorbed on aluminum phosphate with aluminum ≥1.25 mg, ≥10 μg HBsAg and 0.005 thiomersal preservative.

After each vaccination, parents were asked for any complications within 72 hours following the vaccination including erythema or redness of injection site ≥20 mm, general complications including fever ≥38.3 °c, drowsiness, restlessness, persistent crying (more than 3 h), seizure, and anaphylaxis. Infants were also followed for 7 days after vaccination for symptoms of encephalitis. Body temperature was measured with a mercury or digital thermometer.

### Study Assessments

The objective of this study was to determine antibody seroprotection rates (anti-HBs, anti-*Hib* PRP, anti-diphtheria toxoid, antitetanus toxoid, and anti-*Bordetella pertussis*) and calculating geometric mean concentrations (GMC) before vaccination and 4 weeks after the third dose.

Antibody titers were measured by ELISA quantitative kits as follows: Diphtheria (Eurofins Co., with seroprotection defined as antibody concentration <0.01 IU/mL considered as unimmune, 0.01–0.1 IU/mL requiring booster dose and >0.1 IU/mL immune); Tetanus (Eurofins Co., <0.1 IU/mL unimmune, 0.1–1 IU/mL requiring control after 1 to 2 years, 1–5 IU/mL requiring control after 2-4 years and >5 IU/mL requiring control after 4–8 years); Pertussis (Eurofins co., <16 IU/mL unimmune, 16–24 IU/mL borderline and > 24 IU/mL immune); HepB (Diapro Co, <10 IU/mL unimmune, and >10 IU/mL immune); *Hib* (Demeditec Co, <0.15 IU/mL unimmune, and >0.15 IU/mL immune).

Serum isolated from blood samples were stored at a temperature of−20 °c and analyzed after collection.

### Statistical Methods

The cumulative incidence of the outcomes studied (i.e., the adverse events after the Pentavalent immunization) were calculated with 95% confidence intervals (CI). For descriptive statistics, mean, standard deviation and frequency tables were used. Chi-square and Fisher's exact tests were used for analysis. Mean antibody titers and 95% CI for each antibody were calculated and compared before and after vaccination by descriptive statistics and paired *t*-test. All statistical analyses were conducted at a significance level of 0.05 using SPSS software, version 20.0 (SPSS, Chicago, IL, USA).

### Ethical Consideration

Parents of all infants signed written informed consent before enrollment. The study was conducted in accordance with the Declaration of Helsinki and Good Clinical Practice guidelines. The data for the present study was collected from the parents of the children through a questionnaire which was developed for this study. Ethical approval for the present study was obtained from the Ethics Committee of the Mazandaran University of Medical Sciences (IR.MAZUMS.REC.1397.2775).

## Results

**Figure 1:**
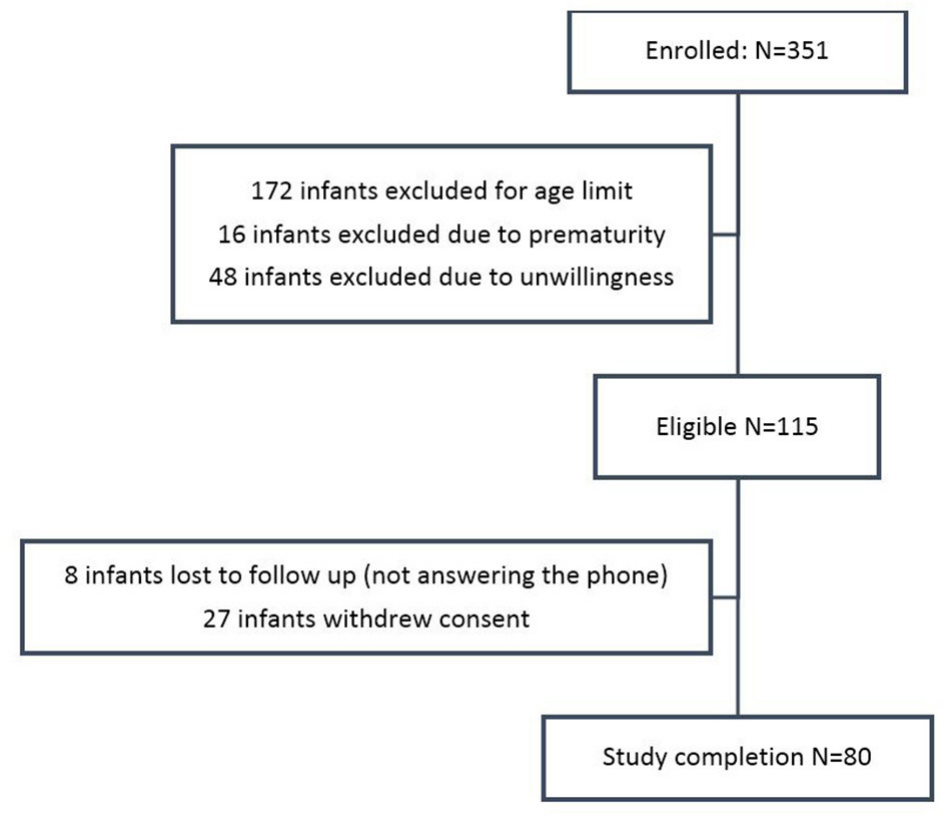
The subject flowchart of the study.

In this study, 115 infants were included, of which 80 infants including 30 girls (37.5%) and 50 boys (62.5%) completed the study. The subject flowchart is shown in Figure 1. Table 1 shows the demographic characteristics of the participants.

**Table 1:** Demographic information of the participants.

	**Variable**	**Percent**	**Number**
Gender	Girl	30	37.5
	Boy	50	62.5
Type of delivery	Normal Vaginal delivery	30	37.5
	Caesarean section	50	62.5
Number of children	1	38	47.5
	2	33	41.3
	3	9	11.3
Admission history	Yes	51	63.8
	No	29	36.3
Underlying disease	Icterus	46	57.5
	Hyper reactive airway	1	1.3
	Urinary tract infection	1	1.3
	Pneumonia	1	1.3
	Cow's milk allergy (CMA)	1	1.3
	Icterus+ pneumonia	1	1.3
	Icterus+ CMA	2	2.5
Anti-pyretic used	Acetaminophen drop	72	90
	Acetaminophen drop+ suppository	6	7.5
	Acetaminophen drop+syrup	0	0
	Acetaminophen drop+Ibuprofen syrup	1	1.3
	Acetaminophen drop+ Diclofenac suppository	1	1.3

The immunogenicity of the Pentavalent vaccine was 100% for tetanus, 98.7% for *Hib* and HepB, 93.1% for diphtheria and 63.7% for pertussis. A comparison of the mean geometric mean titer (GMT) of diphtheria, tetanus, pertussis, HepB and *Hib* before and after the Pentavalent vaccine are shown in Table 2.

**Table 2:** The GMT of Pentavalent vaccine components before and after the study.

	**Immune (%)**	**P-value**	**95% CI**	**GMT (IU/mL)**	**Immune (%)**
Diphtheria	Before	0.22	0.18–0.27	<0.05	72.5
	After	0.43	0.37–0.51		93.8
Tetanus	Before	1.41	1.16–1.74	<0.05	96.2
	After	2.52	2.22–2.88		100
Pertussis	Before	14.30	11.93–17.15	<0.05	41.2
	After	19.44	16.42–23.03		63.7
Hepatitis B	Before	15.25	9.00–25.84	<0.05	36.2
	After	153.54	133.73–176.29		98.7
Hib	Before	0.34	0.28–0.42	<0.05	72.5
	After	2.44	2.06–2.89		98.7

The most common systemic complication following vaccination was fever which occurred in 16.3% after the first vaccination and 20% and 30% after the second and third doses, respectively (*p* = 0.028). Restlessness was the second most common systemic complication, but no significant association was found between restlessness and the Pentavalent vaccine (*p* = 0.348).

Also, 5 infants had erythema at the vaccination site at 2 months of age and 6 and 11 infants experienced this complication after the second and third vaccination, respectively (*p* = 0.107). Only one infant cried for more than 3 h after the second dose. None of the cases reported drowsiness, seizure, encephalitis or anaphylaxis. Table 3 shows the prevalence of complications associated with the Pentavalent vaccine. Also, none of the infants were hospitalized due to vaccination.

**Table 3:** Adverse events associated with Pentavalent vaccination in different ages.

**Side effect**	**2 months *N*(%)**	**4 months *N*(%)**	**6 months *N*(%)**	***P*-value**
Fever	13 (16.3)	16 (20)	24 (30)	0.028
Erythema	5 (6.3)	6 (7.5)	11 (13.8)	0.107
Restlessness	12 (15)	7 (8.8)	10 (12.5)	0.348
Long-term crying	0 (0)	1 (1.3)	0 (0)	0.999
Drowsiness	0 (0)	0 (0)	0 (0)	-
Seizure	0 (0)	0 (0)	0 (0)	-
Anaphylaxis	0 (0)	0 (0)	0 (0)	-
Encephalitis	0 (0)	0 (0)	0 (0)	-
Swelling	0 (0)	0 (0)	0 (0)	-

## Discussion

The ultimate goal of vaccination for public health is to prevent bacterial and viral infections. The Pentavalent vaccine is expected to reduce the procedural steps, vaccination process time, storage needs, and to minimize vaccine wastage as compared to conventional administration with a syringe (2). In the present study, the immunogenicity of the Pentavalent vaccine was evaluated in addition to the comparison of antibody titers before and after three doses and evaluating its complications and side effects in vaccinated infants.

In this study, 1 month after the third dose of Pentavalent vaccine, 79 patients (98.7%) were immune against *Hib*, which was in line with the results of the study by (7). Also, 80% of our study infants had long-time immunity against *Hib* (anti-*Hib* >1 IU/mL) similar to that of study (10). The immunogenicity of the Hexavalent vaccine against *Hib* was reported to be 100% in the study by (11). Also, 95.3% of the infants were immune which could be due to the use of the Radioimmunoassay technique to measure the antibody titer (11). Immunity against *Hib* was 100% in some studies which is slightly higher than our study (12–14). In addition, the number of immune cases was higher in these studies. The difference can be due to the use of different vaccines in these studies.

In our study, 41.2% of the infants were immune against pertussis before vaccination (maternal immunity), which increased to 63.7% 1 month after the third dose of the vaccine. This result was similar to the study of (8). In the study of (15) 6 months after the third dose of the Pentavalent vaccine, 17.3% had pertussis immunity (15). In the study of (16) the immunity in two different centers were 36.7 and 24.6% which was lower than our study. The reason could be the 6-month interval between the third dose of vaccine and titer measurement in (15) study and the use of different measurement kits (16). The anti-pertussis immunity was higher in some studies which could be due to the younger age of infants and shorter intervals between vaccine doses, antibody assay by microagglutination method, higher sample size and different brands of the vaccine (6, 10, 12–14, 17).

In our study, the immunogenicity of hepB component was 98.7%, which was similar to the results of other studies (8, 10, 12, 14, 17) but in (16) study, it was lower than us which could be due to receiving a dose of HepB vaccine at birth in our study population (16).

The immunity against tetanus was 100% in our study which is similar to that of other studies (13, 14, 18, 19) but the results of study were lower (6, 16). The difference may be attributed to vaccination in reproductive age and pregnancy in our study but due to lack of evidence for this in the work, we cannot claim it.

In our study, diphtheria immunity was 93.7% similar to the results of other studies (6, 12, 16). In the study of (18) all cases were immune against diphtheria and had higher immunogenicity compared to us, which could be due to the use of different vaccines (18).

In the present study, the most common systemic complication was fever followed by restlessness and the incidence of fever increased following the second and third doses. The results of other studies were similar to ours (6, 12, 19, 20). In the study of (12) the incidence of fever decreased after the first dose (12). The reason for this difference could be the education of our study parents to properly use a thermometer and using various anti-pyretics with different brands in two studies. The most common systemic complication in the study was restlessness followed by fever (8, 13, 14). The difference with our study could be attributed to different methods of injection, sample size and type of vaccines. The most common complication in the study was restlessness, abnormal crying and drowsiness, respectively which could be due to the hexavalent vaccine and the concomitant use of pneumococcal and meningococcus vaccines (11). (21) reported persistent crying, restlessness and fever as the most common systemic complications which may be due to the use of the Pentavac vaccine (21).

Erythema of the vaccine site was the second common complication in this study which was similar to that of study (13). The most common local complications of the study were pain and erythema of the injection site, respectively (6).

### Study Limitations and Recommendations

The duration of follow-up for evaluating long-term immunity of the vaccine and lack of a control group were the main limitations of this study. Also, evaluating the association between the three doses of pentavalent vaccine was not performed. Therefore, similar studies with long-duration follow-up and evaluating vaccine immunity, larger sample size and simultaneous multicenter studies comparing several vaccines from different companies are recommended. This study demonstrated the Pentalavent vaccine immunity till 18 months of age. Since immunity exists for 18 months, it may justify long term immunity. Further evaluations are needed in 3^rd^ and 4^th^ year of the age.

## Conclusion

The Pentavalent vaccine used in Iran can cause adequate antibody response against diphtheria, tetanus, pertussis, *Hib* and hepatitis B in most cases with minimal side effects. The immunogenicity of this vaccine is significantly lower for pertussis. In this study, no severe complication leading to contraindication to subsequent injections was reported. The present policy in replacing triple DTP vaccine with Pentavalent vaccine which includes hemophilus influenza and hepatitis B should be continued in Iran because it showed a significant impact on community safety, and we expect continuing children's immunization with this vaccine.

## Data Availability Statement

The raw data supporting the conclusions of this article will be made available by the authors, without undue reservation.

## Ethics Statement

The studies involving human participants were reviewed and approved by the Ethics Committee of Mazandaran University of Medical Sciences. Written informed consent to participate in this study was provided by the participants' legal guardian/next of kin.

## Author Contributions

MSR and MJS contributed to the conception and design of the work, and supervision of the study. HaS, SR, and HiS performed the laboratory assessment. ME gathered the data. ANG analyzed the data. FH drafted and edited the manuscript. All authors approved the final content of the submitted version and agree to be accountable for all aspects of the work.

## Conflict of Interest

The authors declare that the research was conducted in the absence of any commercial or financial relationships that could be construed as a potential conflict of interest.

## Publisher's Note

All claims expressed in this article are solely those of the authors and do not necessarily represent those of their affiliated organizations, or those of the publisher, the editors and the reviewers. Any product that may be evaluated in this article, or claim that may be made by its manufacturer, is not guaranteed or endorsed by the publisher.

## Abbreviations

CI: Confidence interval
CMA: Cow's milk allergy
EPI: Expanded Program on Immunization
GMT: Geometric mean titer
HepB: Hepatitis B
Hib: *Haemophilus influenzae type b*
WHO: World Health Organization.
